# Supplementary material for: Staff-Facilitated Telemedicine Care Delivery for Treatment of Hepatitis C Infection among People Who Inject Drugs
Source: Healthcare (Basel). 2024 Mar 25;12(7):715. doi: 10.3390/healthcare12070715 (PMC11012066; doi:10.3390/healthcare12070715)
Supplement: Supplementary file 1 [file healthcare-12-00715-s001.zip › healthcare-2863209-supplementary.pdf]

NOW Study Telemedicine Questionnaire

TEL01 In the **past month**, have you ***used*** any of the following devices?

[Note: this question does not ask about ownership, just "use".]

	Yes	No
Basic landline telephone	○	○
Basic cell phone (without internet browsing capabilities)	○	○
Smartphone (iPhone, Android, Blackberry, Google Pixel) [Note: we define a "smartphone" as a phone that has a touchscreen, internet access, and an operating system]	○	○
Tablet with internet browsing capabilities (iPad, Samsung Galaxy Tab, Motorola Xoom, Amazon Fire)	○	○
Computer/laptop	○	○

TEL02 Is the [each device below] that you used "your own", "a friends/family member's", or "from a public space"?

	Your own	A friend/family's	From a public space (library, coffee shop)
Basic cellular telephone (flip phone)	○	○	○
Smartphone (iPhone, Android, Blackberry, Google Pixel) [Note: we define a "smartphone" as a phone that has a touchscreen, internet access, and an operating system].	○	○	○
Tablet computer with internet/WiFi capabilities (iPad, Samsung Galaxy Tab, Motorola Xoom, Amazon Fire)	○	○	○
Computer/laptop	○	○	○

TEL05 In the PAST WEEK, how often did you go online or use the internet?

○ Multiple times a day

○ Once per day

○ More than half of the days (4-6 days)

○ Less than half of the days (1-3 days)

○ **I have not accessed internet or wifi in the past week**

Start of Block: TELEMED_2

TEL31 Overall, how was your experience receiving clinical care via telemedicine (video streamed conversation) during the NOW Study?

- ◯ Extremely helpful
- ◯ Somewhat helpful
- ◯ Somewhat unhelpful
- ◯ Extremely unhelpful
- ◯ Decline

TEL32 What things did you **like** about receiving clinical care via telemedicine during the NOW Study?

____

____

____

____

____

TEL33 What things did you **NOT** like about receiving clinical care via telemedicine during the NOW Study?

____

____

____

____

____

TEL34 How did your experience receiving clinical care via telemedicine during the NOW Study compare to your **other experiences with telemedicine,** if any?

- NOW study telemedicine was a lot better
- NOW study telemedicine was a little better
- NOW study telemedicine was a little worse
- NOW study telemedicine was a lot worse
- **I haven’t participated in telemedicine outside of the NOW Study**
- Decline

TEL27 How interested are you in using telemedicine to address other health priorities?

- Very interested
- Interested
- Uninterested
- Very uninterested
- Decline

TEL35 What is a current health priority you would feel comfortable using telemedicine to address? [Pause]

- ☐ Primary care
- ☐ Mental health care/therapy
- ☐ Hypertension
- ☐ Diabetes
- ☐ Heart failure
- ☐ COPD/asthma
- ☐ Liver failure/cirrhosis
- ☐ Women’s health/gynecology
- ☐ Substance use treatment
- ☐ Chronic pain
- ☐ Arthritis
- ☐ Wound care
- ☐ HIV
- ☐ Other: ____

TEL36 What is a current health priority you would **NOT** feel comfortable using telemedicine to address? [Pause]

- ☐ Primary care
- ☐ Mental health care/therapy
- ☐ Hypertension
- ☐ Diabetes
- ☐ Heart failure
- ☐ COPD/asthma
- ☐ Liver failure/cirrhosis
- ☐ Women’s health/gynecology
- ☐ Substance use treatment
- ☐ Chronic pain
- ☐ Arthritis
- ☐ Wound care
- ☐ HIV
- ☐ Other: ________________________________

Page Break

TEL37 In your own words, what did you think about your clinical care provided during the NOW Study?

____

____

____

____

____

TEL38 Please rate the level to which you agree or disagree with the following statements about your telemedicine **video visit(s)**. Answer options include “strongly disagree”, “disagree”, “agree”, and “strongly agree”.

	Strongly agree	Agree	Disagree	Strongly disagree	Decline
I thought the telemedicine visit was easy to do.	○	○	○	○	○
The doctor answered my questions and provided useful information about my Hepatitis C care.	○	○	○	○	○
I felt comfortable asking questions and discussing medical issues.	○	○	○	○	○
I was able to discuss my medical concerns/problems well enough during the video visit.	○	○	○	○	○
I was worried about discussing confidential or personal topics.	○	○	○	○	○
I would have received better clinical care during the NOW Study if you saw the clinician in person (instead of via video visit).	○	○	○	○	○

TEL28 Overall, if you had the option would you prefer to see your healthcare provider by telemedicine visit (telephone or video visit) or in person at a clinic?

- ○ Telemedicine visit - by telephone
- ○ Telemedicine visit - by video
- ○ In person at clinic

TEL29 What are all of your **concerns** related to communicating with a healthcare provider by **video** visit instead of in the clinic?

- ☐ Don’t have a phone or computer for the visit
- ☐ Reasons related to phone plan or internet quality (e.g., not enough minutes/data, poor internet quality)
- ☐ Not feeling comfortable with the technology
- ☐ Preferred in-person visit at clinic
- ☐ Didn’t feel comfortable talking about medical topics over phone/online
- ☐ Didn’t have a private place to conduct visit
- ☐ Other ____
- ☐ **I had no concerns about the video visit.**

TEL39 Now I am going to ask you to rate each item that you noted on a scale of 1-10 based on your level of concern; with one being the lowest level of concern and 10 being the highest level of concern.

	0 1 2 3 4 5 6 7 8 9 10
Don’t have a phone or computer for the visit	
Reasons related to phone plan or internet quality (e.g., not enough minutes/data, poor internet quality)	
Not feeling comfortable with the technology	
Preferred in-person visit at clinic	
Didn’t feel comfortable talking about medical topics over phone/online	
Didn’t have a private place to conduct visit	
Other	
**I had no concerns about the video visit.**	
